# Case Report: Application of tumor-bearing bone inactivation and bilateral fibula grafting in joint-sparing surgery for osteosarcoma patient

**DOI:** 10.3389/fsurg.2025.1544336

**Published:** 2025-04-29

**Authors:** Yapeng Wang, Tian’en Xu, Cong Chen, Shuo Mai, Kai Yang

**Affiliations:** The Department of Orthopedics, The Second Hospital & Clinical Medical School, Lanzhou University, Lanzhou, Gansu, China

**Keywords:** osteosarcoma, tumor-bearing bone inactivation, autologous fibula grafting, joint-sparing surgery, bone defect reconstruction

## Abstract

On August 28th, 2023, a 13-year-old male was diagnosed with conventional osteosarcoma of the proximal left tibia after a needle biopsy. Subsequently, the patient received two cycles of neoadjuvant chemotherapy and four cycles of postoperative chemotherapy. On December 27, 2023, the tumor resection was performed while preserving the knee joint, which involved inactivation of the tumor-bearing bone, autologous bilateral fibula grafting, and fixation of the grafted bone to the host bone using plate and screws. Follow-up after surgery included x-rays and CT scans. On February 28, 2024, two months after the surgery, new bone formation was noted at the site from which bone was harvested from the right fibula, the left knee joint had satisfactory range of motion in flexion (130°) and extension (0°). Additionally, partial healing of both the grafted bone and the host bone was observed. In the follow-up on September 23rd, 2024, nine months post-operation, the right fibula had reformed. Furthermore, the transplanted and host bones of the left tibia had healed securely. It was confirmed that there was no recurrence or metastasis of the tumor during the last follow-up by ECT. This case highlights the feasibility and effectiveness of using inactivating tumor-bearing bone and autologous bilateral fibular grafting to repair large bone defects after joint-sparing surgery for malignant tumors near the joints.

## Introduction

Osteosarcoma is a prevalent and aggressive bone tumor that mainly affects adolescents and young adults. The standard treatment usually includes neoadjuvant chemotherapy, surgery, and follow-up adjuvant chemotherapy. Various reconstruction techniques for bone defects after surgery include hinged prosthetic arthroplasty (like modular and 3D-printed prostheses) (1, 2), allogeneic bone grafting (3), replantation of inactivated tumor-bearing bone (4), autologous bone grafting (5), and Ilizarov external fixator-assisted bone transport (6). Despite these treatment options, minimizing complications, achieving satisfactory functional outcomes, and preventing tumor recurrence and metastasis remain major challenges. In this context, exploring new and effective treatment methods is critically important.

## Case presentation

**Figure 1:**
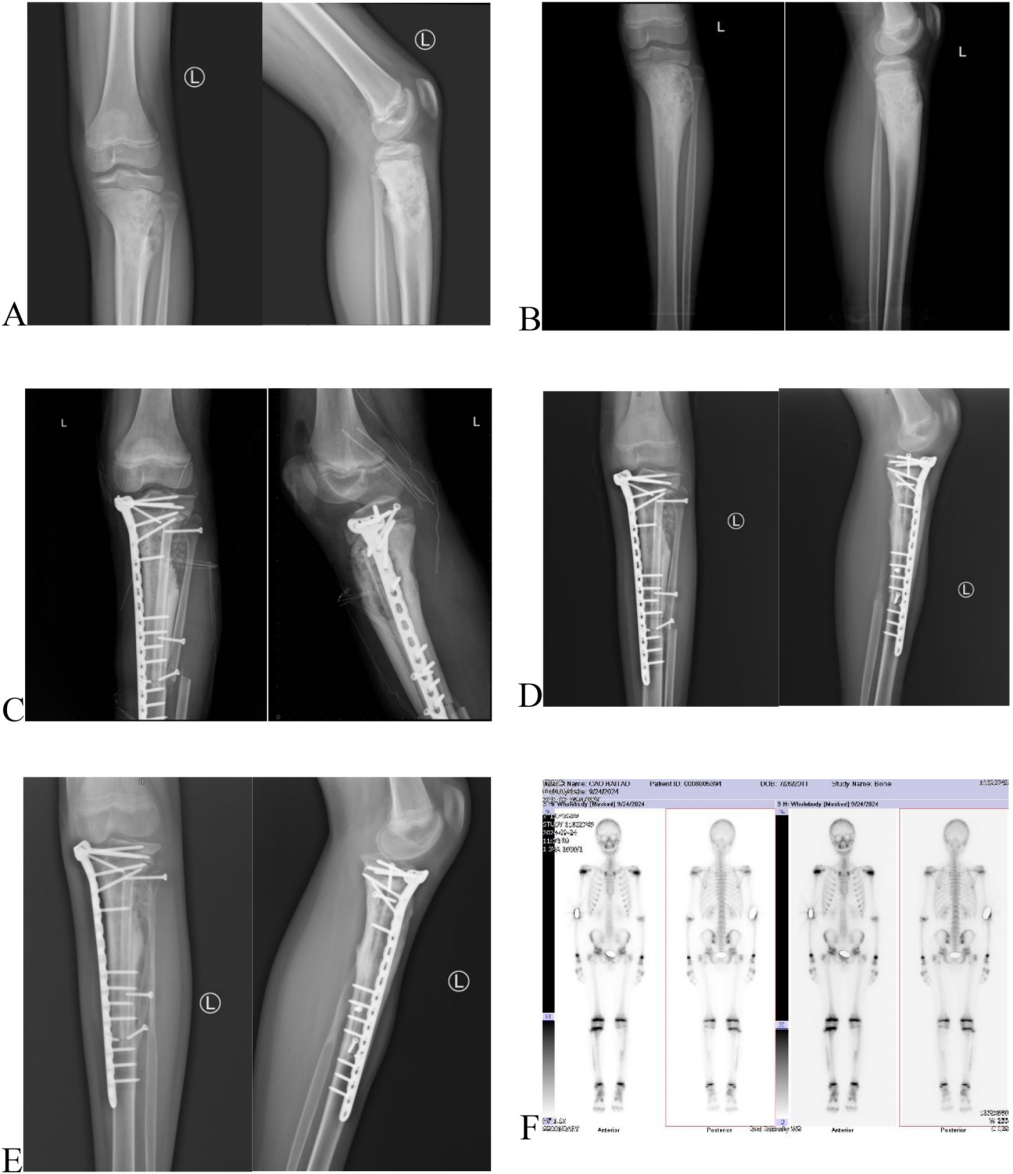
**(A)** The x-ray before neoadjuvant chemotherapy: bone destruction and periosteal reaction in the proximal left tibia. **(B)** Preoperative x-ray of the left tibia following neoadjuvant chemotherapy: Osteosarcoma of the left tibia. **(C)** Postoperative x-ray of the left tibia within 24 h after surgery: Tumor-bearing bone inactivation and replantation, autologous bilateral fibula grafting with plate and screws internal fixation. **(D)** Two-month postoperative x-ray of the left tibia: Partial osseous union between the composite graft and the host bone. **(E)** Nine-month postoperative x-ray of the left tibia: Complete osseous union between the composite graft and the host bone. F Nine-month postoperative ECT: No evidence of tumor recurrence.

**Figure 2:**
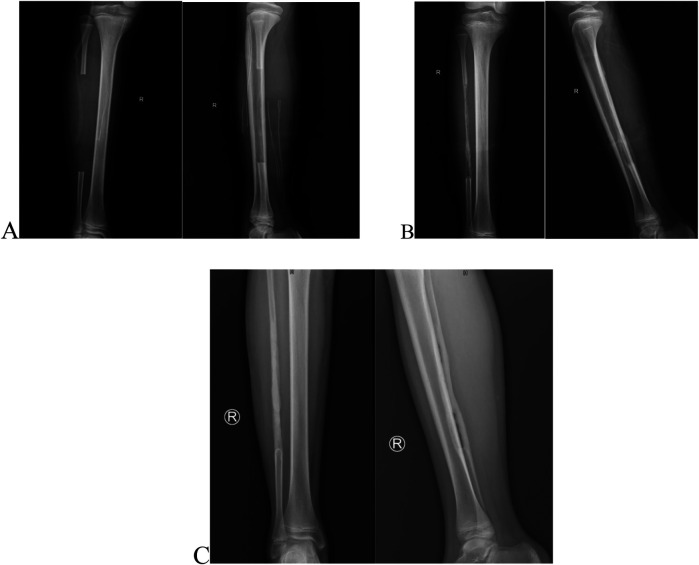
**(A)** Postoperative x-ray of the right tibia within 24 h after surgery: segmental fibular resection with periosteal preservation. **(B)** Two-month postoperative x-ray of the right tibia: Partial osteogenesis at the right fibula. **(C)** Nine-month postoperative x-ray of the right tibia: Extensive osteogenesis was observed in the right fibula, yet the medullary cavity was not fully formed.

**Figure 3:**
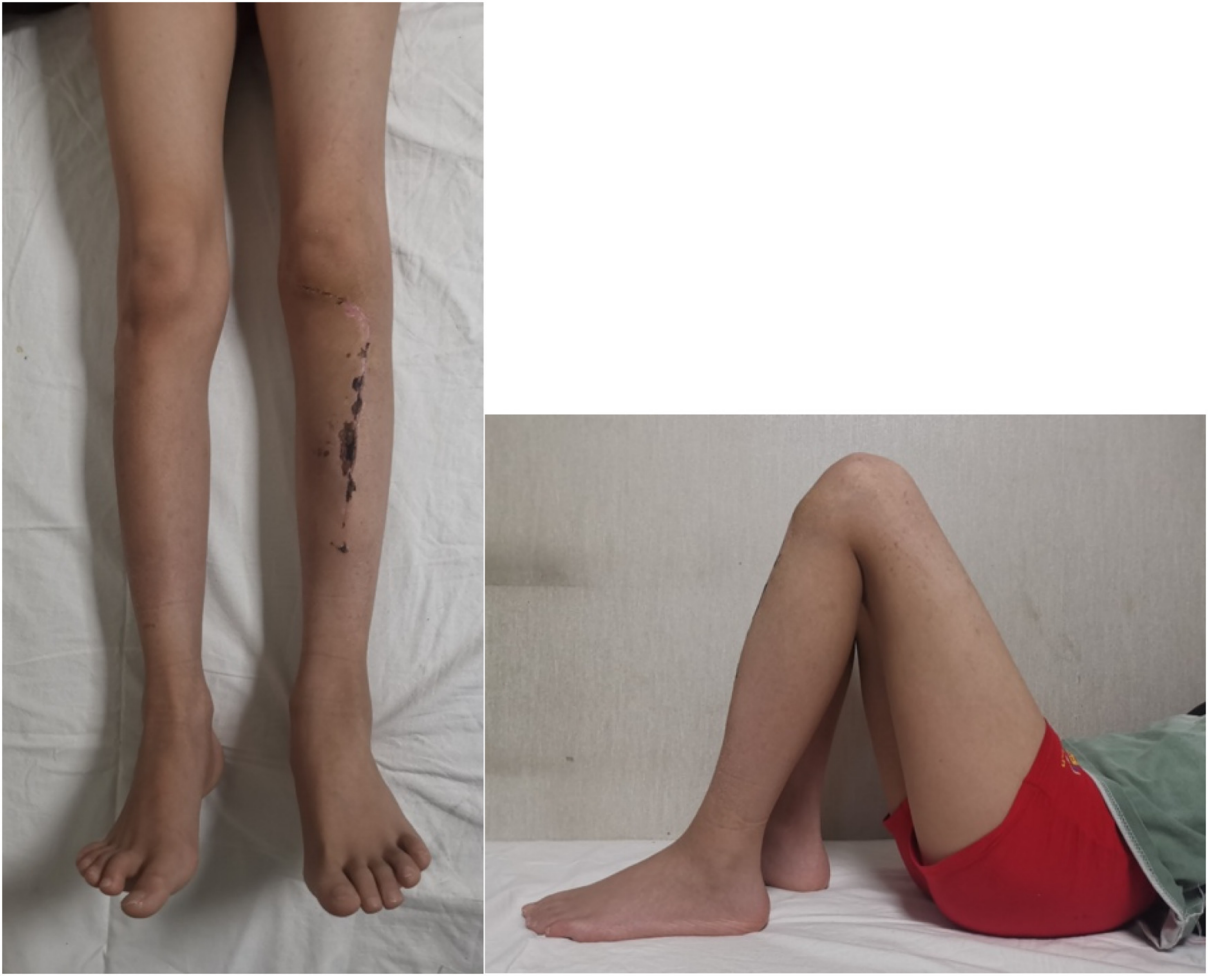
Two-month post-operation, the flexion of the knee joint was 130°, and the extension was 0°.

This case involves a 13-year-old boy who has experienced swelling and pain in left knee for two months, and after a thorough evaluation, he was diagnosed with conventional osteosarcoma via a needle biopsy (Figure 1A). Prior to the surgery, the patient received one cycle of neoadjuvant chemotherapy that included epirubicin, cisplatin, and methotrexate. Following this, the patient underwent another cycle containing epirubicin, ifosfamide, and methotrexate. After receiving neoadjuvant chemotherapy, the patient underwent joint-preserving surgery (Figures 1B, 2A). Then the operation used an innovative method that combined the inactivation of tumor segmental bone with grafting of the patient's own bilateral fibulas. During the surgery, the tumor-bearing bone was immersed in 10% sodium chloride solution at 65 degrees Celsius for 30 min to inactivate, the right free fibula was harvested and placed into the medullary cavity of the inactivating tumor bone. Simultaneously, the left vascularized fibula underwent an osteotomy at the tibial plane, was then moved medially, and had the surface of the proximal tibiofibular joint excised, then secured to the host bone with plate and screws (Figure 1C). Four weeks after surgery, the patient wore a lower limb brace and performed non-weight-bearing activities, and one week later, the patient initiated four cycles of adjuvant chemotherapy, which included epirubicin, ifosfamide, and methotrexate. By 12 weeks after surgery, the patient advanced to weight-bearing activities. And postoperative follow-up was carried out through x-ray and CT scans. On February 28, 2024, two months post-surgery, new bone formation was observed in the right tibia (Figure 2B), along with partial healing of the transplanted and host bones (Figure 1D) and the knee joint demonstrated good flexion (130°) and extension (0°) (Figure 3). By September 23, 2024, nine months post-operation, the right fibula had remodeled (Figure 2C), the transplanted and host bones of the left tibia had healed firmly (Figure 1E), and ECT showed no evidence of tumor recurrence (Figure 1F).

## Discussion

Studies have examined the effectiveness of various limb-salvage techniques. However, each reconstruction method has distinct benefits and drawbacks in terms of effectiveness and complications. For instance, prosthetic replacement can quickly restore function but carries long-term risks, including loosening and infection (2, 7). Inactivation and replantation of tumor-bearing bone may result in longer healing times and non-union (8). Allogeneic bone grafting could encounter complications such as immune rejection and infection (3, 7). Autologous bone grafting avoids immune rejection but has challenges such as donor site complications and limited bone volume (5). And the Ilizarov external fixator-assisted bone transport may lead to complications, including infections, delayed unions, and joint stiffness (9, 10). The innovative combination of tumor-bearing bone inactivation and autologous bilateral fibula grafting not only achieved excellent reconstruction of the proximal tibia but also improved the integration of the graft with the host bone. In this case, the contralateral free fibula graft was selected due to its multiple key benefits. Firstly, the inactivated bone exhibited significant defects and substantial bone loss. This made it crucial to provide additional structural support along with a dependable bone grafting function. Secondly, the extensive resection of the tumor left no suitable vessels for anastomosis in the surgical area. In pediatric patients, managing large bone defects is challenging, and traditional techniques often result in poor functional recovery and increased morbidity. This method presents an innovative solution to effectively resolve these issues.

In this case, Preserving the right fibula periosteum during the surgical procedure likely promoted the observed new bone formation. This phenomenon was also noted by Colangeli (11). However, the absence of medullary cavity formation is still concerning. This could be due to the characteristics of the newly formed bone, which might lack the structural properties needed for tubular bone formation, or it may be a result of the relatively short follow-up period. Future studies should focus on the long-term outcomes of these surgical techniques and investigate methods to enhance medullary cavity formation through adjunctive therapies.

## Conclusion

This case demonstrates the promise of combining tumor-bearing bone inactivation with autologous bilateral fibula grafting as an effective strategy for managing bone defects caused by tumors. The successful maintenance of knee joint function and early signs of bone healing suggest that this method could be a valuable addition to the surgical options available for treating osteosarcoma in children. Further research is needed to refine these techniques and create standardized protocols for their use in clinical practice.

## Data Availability

The original contributions presented in the study are included in the article/Supplementary Material, further inquiries can be directed to the corresponding author.

## References

[1] Mayfield CK Ayad M Lechtholz-Zey E Chen Y Lieberman JR. 3D-printing For critical sized bone defects: current concepts and future directions. Bioengineering (Basel). (2022) 9(11):680. 10.3390/bioengineering9110680 36421080 PMC9687148

[2] Ebeid WA Hassan MHA. Functional outcome following proximal tibial osteosarcoma resection and reconstruction by modular endoprosthesis. Ann Surg Oncol. (2023) 30(3):1914–25. 10.1245/s10434-022-12788-3 36437409 PMC9908643

[3] Aponte-Tinao L Ayerza MA Muscolo DL Farfalli GL. Survival, recurrence, and function after epiphyseal preservation and allograft reconstruction in osteosarcoma of the knee. Clin Orthop Relat Res. (2015) 473(5):1789–96. 10.1007/s11999-014-4028-5 25352262 PMC4385338

[4] Dai Z Sun Y Maihemuti M Jiang R. Follow-up of biological reconstruction of epiphysis preserving osteosarcoma around the knee in children: a retrospective cohort study. Medicine (Baltimore). (2023) 102(10):e33237. 10.1097/MD.0000000000033237 36897729 PMC9997815

[5] Campanacci DA Scanferla R Marsico M Scolari F Scoccianti G Beltrami G Intercalary resection of the tibia for primary bone tumors: are vascularized fibula autografts with or without allografts a durable reconstruction? Clin Orthop Relat Res. (2024) 482(6):960–75. 10.1097/CORR.0000000000003007 38513152 PMC11124688

[6] Wang W Yang J Wang Y Han G Jia JP Xu M Bone transport using the Ilizarov method for osteosarcoma patients with tumor resection and neoadjuvant chemotherapy. J Bone Oncol. (2019) 16:100224. 10.1016/j.jbo.2019.100224 30989037 PMC6447741

[7] Albergo JI Gaston CL Aponte-Tinao LA Ayerza MA Muscolo DL Farfalli GL Proximal tibia reconstruction after bone tumor resection: are survivorship and outcomes of endoprosthetic replacement and osteoarticular allograft similar? Clin Orthop Relat Res. (2017) 475(3):676–82. 10.1007/s11999-016-4843-y 27103142 PMC5289179

[8] Takeuchi A Tsuchiya H Setsu N Gokita T Tome Y Asano N What are the complications, function, and survival of tumor-devitalized autografts used in patients with limb-sparing surgery for bone and soft tissue tumors? A Japanese musculoskeletal oncology group multi-institutional study. Clin Orthop Relat Res. (2023) 481(11):2110–24. 10.1097/CORR.0000000000002720 37314384 PMC10566762

[9] Aktuglu K Erol K Vahabi A. Ilizarov bone transport and treatment of critical-sized tibial bone defects: a narrative review. J Orthop Traumatol. (2019) 20(1):22. 10.1186/s10195-019-0527-1 30993461 PMC6468024

[10] Feng D Zhang Y Jia H Xu G Wu W Yang F Complications analysis of Ilizarov bone transport technique in the treatment of tibial bone defects-a retrospective study of 199 cases. BMC Musculoskelet Disord. (2023) 24(1):864. 10.1186/s12891-023-06955-0 37936087 PMC10629116

[11] Colangeli M Spinnato P Manfrini M. Periosteum preservation in bone regeneration. CMAJ. (2020) 192(32):E920. 10.1503/cmaj.200005 32778605 PMC7829018

